# Combining active learning and semi-supervised learning techniques to extract protein interaction sentences

**DOI:** 10.1186/1471-2105-12-S12-S4

**Published:** 2011-11-24

**Authors:** Min Song, Hwanjo Yu, Wook-Shin Han

**Affiliations:** 1 Information Systems Department, New Jersey Institute of Technology, University Heights, Newark, New Jersey, USA; 2 Department of Computer Science & Engineering, POSTECH, Pohang, South Korea; 3 School of IT Engineering, Kyungpook National University, Daegu, South Korea

## Abstract

**Background:**

Protein-protein interaction (PPI) extraction has been a focal point of many biomedical research and database curation tools. Both Active Learning and Semi-supervised SVMs have recently been applied to extract PPI automatically. In this paper, we explore combining the AL with the SSL to improve the performance of the PPI task.

**Methods:**

We propose a novel PPI extraction technique called PPISpotter by combining Deterministic Annealing-based SSL and an AL technique to extract protein-protein interaction. In addition, we extract a comprehensive set of features from MEDLINE records by Natural Language Processing (NLP) techniques, which further improve the SVM classifiers. In our feature selection technique, syntactic, semantic, and lexical properties of text are incorporated into feature selection that boosts the system performance significantly.

**Results:**

By conducting experiments with three different PPI corpuses, we show that PPISpotter is superior to the other techniques incorporated into semi-supervised SVMs such as Random Sampling, Clustering, and Transductive SVMs by precision, recall, and F-measure.

**Conclusions:**

Our system is a novel, state-of-the-art technique for efficiently extracting protein-protein interaction pairs.

## Background

Automated protein-protein interaction (PPI) extraction from unstructured text collections is a task of significant interest in the bio-literature mining field. The most commonly addressed problem has been the extraction of binary interactions, where the system identifies which protein pairs in a sentence have a biologically relevant relationship between them. Proposed solutions include both hand-crafted rule-based systems and machine learning approaches [1]. Recently Semi-supervised Learning (SSL) techniques have been applied to PPI tasks [2]. SSL is a Machine Learning (ML) approach that combines supervised and unsupervised learning where typically a small amount of labeled and a large amount of unlabeled data are used for training. SSL has gained significant attention to PPI extraction because of two reasons. First, labeling of a large set of instances is labor-intensive and time-consuming. This task has to be also carried out by qualified experts and thus is expensive. Second, several studies show that using unlabeled data for learning improves the accuracy of classifiers [3,4].

One major problem of SSL is that it may introduce incorrect labels to the training data, as the labeling is done by machine, and such labeling errors are critical to the classification performance. Active Learning (AL) can complement the SSL by reducing such labeling errors [5]. AL is a technique of selecting a small sample from the unlabeled data such that labeling on the sample maximizes the learning accuracy. The selected sample is manually labeled by experts. In this paper, we explore combining the AL with the SSL to improve the performance of the PPI task. To our best knowledge, this is the first attempt to apply a combination of semi-supervised and active learning for the extraction task of protein-protein interaction.

The contributions of this paper are three fold. First, we proposed a novel PPI extraction technique called PPISpotter by combining Deterministic Annealing-based SSL and an AL technique to extract protein-protein interaction. Second, we extracted a comprehensive set of features from MEDLINE records by Natural Language Processing (NLP) techniques, which further improve the SVM classifiers. In our feature selection technique, syntactic, semantic, and lexical properties of text are incorporated into feature selection that boosts the system performance significantly. Third, we conducted experiments with three different PPI corpuses and showed that PPISpotter is superior to other techniques by precision, recall, and F-measure.

Many approaches have been proposed to extract protein-protein interaction from unstructured text. One approach employs pre-specified patterns and rules for PPI extraction [6]. However, this approach is often inapplicable to complex cases not covered by the pre-defined patterns and rules. Huang et al. [7] proposed a method where patterns are discovered automatically from a set of sentences by dynamic programming.

The second approach utilizes dictionary. Blaschke et al. [8] extracted protein-protein interactions based on co-occurrence of the form “… p1…I1… p2” within a sentence, where p1, p2 are proteins and I1 is an interaction term. Protein names and interaction terms (e.g., activate, bind, inhibit) are provided as a “dictionary.” Pustejovsky et al. [9] extracted an “inhibit” relation for the gene entity from MEDLINE. Jenssen et al. [10] extracted gene-gene relations based on co-occurrence of the form “… g1…g2…” within a MEDLINE abstracts, where g1 and g2 are gene names. Gene names were provided as a “dictionary”, harvested from HUGO, LocusLink, and other sources. Although their study uses 13,712 named human genes and millions of MEDLINE abstracts, no extensive quantitative results are reported and analyzed. Friedman et al. [11] extracted a pathway relation for various biological entities from a variety of articles.

The third approach is based on machine learning techniques. Bunescu et al. [1] conducted protein/protein interaction identification with several learning methods such as pattern matching rule induction (RAPIER), boosted wrapper induction (BWI), and extraction using longest common subsequences (ELCS). ELCS automatically learns rules for extracting protein interactions using a bottom-up approach. They conducted experiments in two ways; one with manually crafted protein names and the other with the extracted protein names by their name identification method. In both experiments, Zhou et al. [12] proposed two novel semi-supervised learning approaches, one based on classification and the other based on expectation-maximization, to train the HVS model from both annotated and un-annotated corpora. Song et al. [13] utilized syntactical, as well as semantic cues, of input sentences. By combining the text chunking technique and Mixture Hidden Markov Models, They took advantage of sentence structures and patterns embedded in plain English sentences. Temkin and Gilder [14] used a full parser with a lexical analyzer and a context free grammar (CFG) to extract protein-protein interaction from text. Alternatively, Yakushiji et al. [15] propose a system based on head-driven phrase structure grammar (HPSG). In their system protein interaction expressions are presented as predicate argument structure patterns from the HPSG parser. These parsing approaches consider only syntactic properties of the sentences and do not take into account semantic properties. Thus, although they are complicated and require many resources, their performance is not satisfactory. Mitsumori et al. [16] used SVM to extract protein-protein interactions. They use bag-of-words features, specifically the words around the protein names. These systems do not use any syntactic or semantic information. Miyao et al. [17] conducted a comparative evaluation of several state-of-the-art natural language parsers, focusing on the task of extracting protein–protein interaction (PPI) from biomedical papers. They found marginal difference in terms of accuracy but more significant differences in parsing speed. BioPPISVMExtractor is a recent PPI extraction system developed with SVM [2]. It utilizes rich feature sets such as word features, keyword feature, protein names distance feature, and Link Grammar extraction results for protein-protein interaction extraction. They observed that the rich feature sets help improve recall at the cost of a moderate decline in precision.

Cui et al. [18] applied an uncertainty sampling based method of active learning for a lexical feature-based SVM model to tag the most informative unlabeled samples. They reported that the performance of the active learning-based technique on AIMED and CB corpora was significantly improved in terms of reduction of labelling cost.

## Methods

**Figure 1:**
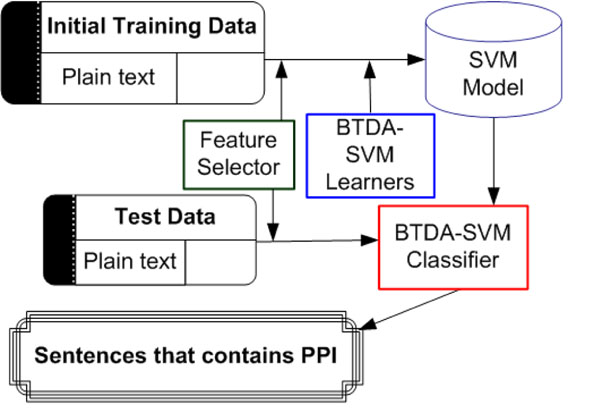
System architecture of PPISpotter

In this section, we describe the overall architecture and procedures of PPISpotter (Figure 1). PPISpotter incorporates AL models into SSL SVMs for extraction of protein-protein interaction. PPISpotter also automatically converts a sentence into 9 feature sets based on the technique described in Section 4.

Below is a set of steps that PPISpotter processes.

**Step 1:** Preprocess the initial training data. The feature selector applies the feature selection technique proposed in Section 4 to the preprocessed data sets.

**Figure 2:**
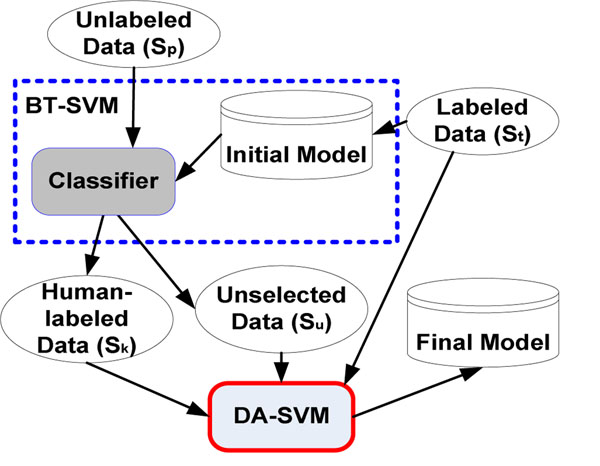
Combination of active learning with semi-supervised learning

**Step 2:** Train the model. Two classifiers, Break Tie-based SVM (BT-SVM) and Deterministic Annealing-based SVM (DA-SVM) classifiers are combined to train the model (a.k.a. BTDA-SVM). Figure 2 illustrates how to combine these two techniques (Blue dot line is the BT-SVM procedure and red solid line is the DA-SVM procedure). At this stage, the human expert provides feedback to the system for a set of instances in the fuzzy unlabeled data. Note that the BT-SVM classifier is based on the Break Tie active learning approach and DA-SVM classifier is based on the Deterministic Annealing technique.

**Step 3:** Take the input data and convert it to the same format as the training data. The feature selector performs the same task as in Step 1.

**Step 4:** Apply the BTDA-SVM learner to identify sentences that contain protein-protein interaction.

**Step 5:** Store extracted sentences to the database.

### Combination of active learning with semi-supervised learning

One of the goals of this paper is to combine SSL and AL into a unified semi-supervised active learning technique for protein-protein interaction extraction. We employ a proportion of unlabeled data in the learning tasks in order to resolve the problem of insufficient training data.

Our strategy of combining AL with SSL is inspired by the Tur et al.’s study [5]. We employ the break tie AL technique (BT-SVM) to train a classifier on both labeled and unlabeled data, and return to the user the most relevant results. Then, the learning system trains a classifier based on the Deterministic Annealing SSL technique (DA-SVM) on both the labeled and unlabeled data (S_t_, S_k_, and S_u_), and results in the final model (Figure 2).

**Figure 3:**
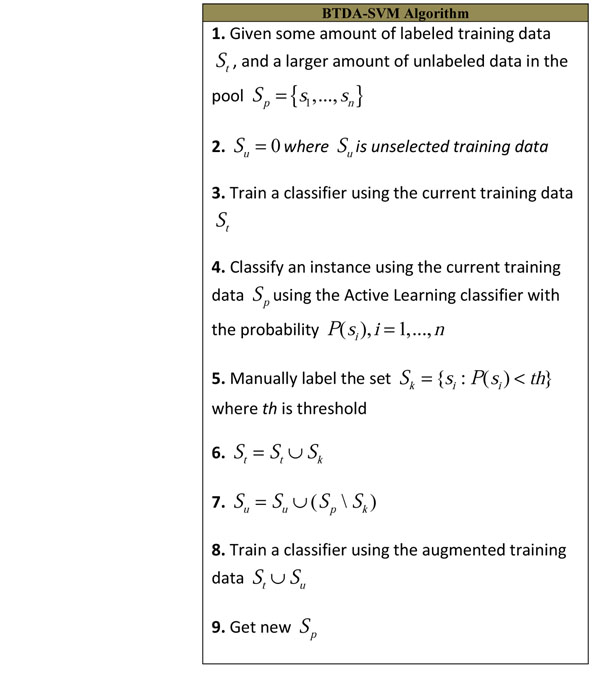
BTDA-SVM algorithm

BTDA-SVM is a combination of the active learning algorithm presented in Section 4 and the semi-supervised learning algorithm presented in Section 5. Instead of leaving out the instances classified with high confidence scores, this algorithm exploits them. Figure 3 explains the BTDA-SVM algorithm.

### Active learning

Active learning, known as pool-based active learning, is an interactive learning technique designed to reduce the labor cost of labeling in which the learning algorithm can freely assign the unlabeled data instances to the training set. The basic idea is to select the most informative data instances for labeling by the users in the next learning round. In other words, the strategy of active learning is to select an optimal set of unlabeled data instances that minimizes the expected risk of the next round.

#### Breaking tie (BT)

For a given instance, the regular SVMs results in distances among instances whose range is from 0 to 1. The value 0 means that the instance lies on the hyperplane and the value 1 indicates that the instance is a support vector.

To assign a probability value to a class the sigmoid function can be used with the assumption that a probability associated with a classifier indicates to which extent the classification result is trusted. In this case, Luo et al. [19] defines the parametric model in the following form:(1)

where *A* and *B* are scalar values, which have to be estimated and *f* is the decision function of the SVMs. This parametric model is used for calculating the probabilities. To use this model, the SVM parameters (complexity parameter *C*, kernel parameter *k*) and the parameter *A* and *B* need to be calculated. Although cross validation can be used for this calculation, it is computationally expensive. An alternative is a pragmatic approximation method that all binary SVMs have the same *A* while eliminating *B* by assigning 0.5 to instances lying on the decision boundary and by trying to compute the SVM parameters and *A* simultaneously [19].

The decision function can be normalized by its margin to include the margin in the calculation of the probabilities.(2)

where we currently look at class *p* and *P_pq_* is the probability of class *p* versus class *q*. We assume that *P_pq_*, q=*1*,2,... are independent. The final probability for class *p*:(3)

It has been reported that the performance bases on this approximation is fast and accurate [19]. This probability model serves as basis for the Breaking Tie algorithm for semi-supervised learning.

### Semi-supervised support vector machines

Support Vector Machines (SVMs) is a supervised machine learning approach designed for solving two-class pattern recognition problems. SVMs adopts maximum margin to find the decision surface that separates the positive and negative labeled training examples of a class [20].

Transductive Support Vector Machines (TSVMs) is an extended version of SVM that uses unlabeled data in addition to labeled data for train classifiers [21]. The goal of TSVMs is to determine which test data instances result in the maximum-margin hyperplane that separates the positive and negative examples for classifiers. Since every test instances need to be included in the SVM’s objective function, finding the exact solution to the resulting optimization problem is intractable. To resolve this issue, Joachims [21] proposed an approximation algorithm. One issue of Joachims’ approach, however, is that it requires the similar distribution of positive and negative instances between the test data and the training data. This requirement is difficult to meet particularly when the training data is small. The challenge is to find a decision surface that separates the positive and negative instances of the original labeled data and the unlabeled data to unlabeled data to be converted to labeled data with maximum margin. The unlabeled data sets apart the decision boundary from the dense regions, and the optimization problem is NP-hard [22]. Various approximation algorithms are found in [22].

The optimization problem held in TSVMs is a non-linear non-convex optimization [23]. Past several years, researchers have attempted to solve this critical problem. Chapell and Zien [24] proposed a smooth loss function, and a gradient descent method to find the decision boundary in a region of low density. Another technique is a branch-and-bound method [25] that searches for the optimal solution. But, it is applicable to a small number of examples due to involving the heavy computational cost. Despite the success of TSVM, the unlabeled data does not necessarily improve classification accuracy.

As an alternative to TSVMs, we explore an Deterministic Annealing approach to semi-supervised SVMs. The first approach was proposed by Luo and his colleagues [19] that formulated a probabilistic framework for image recognition. The Deterministic Annealing (DA) approach is the second proposed by Sindhwani et al. [26]. In the probabilistic framework, semi-supervised learning can be modeled as a missing data problem, which can be addressed by generative models such as mixture models. In the case of semi-supervised learning, probabilistic approaches provide us with various different ways to query unlabeled instances for labeling. A simple method is to train a model on the given labeled datasets and use this model on the unlabeled data. Each of these unlabeled instances is given probabilities that these instances belong to a given class. We can query the least certain instances or the most certain instances. The detailed description of the Deterministic Annealing semi-supervising learning is provided in the study of Luo and his colleagues [19].

#### Deterministic annealing (DA)

Deterministic annealing (DA) is a special case of a homotopy method for combinatorial optimization problems [26]. We adopt the DA technique proposed by Sindhwani et al. [26] to extraction of protein-protein interaction. The detailed description of applying DA for SVMs is provided by Sindhwani et al. [26].

Suppose one is given a following non-convex optimization problem:(4)

DA finds a local minimum of this in the following: First, DA treats the discrete variables as random binary variables over a space of probability distributions *P*. Second, to solve the optimization problem, DA finds a distribution *p ∈ P* that minimizes the expected value of *F*. It makes the optimization problem to be continuous. For this reason, an additional convex term is added to the objective function which is the entropy *S* of the distribution denoted in Eq. 1.(5)

where the parameter *T* controls the trade-off between the expectation and the entropy (called the temperature of the problem) and *y* ∈ {0,1}*^n^* are the discrete variables for the objective function *F*(*y*). For *T* = *0* and *P* including all point-mass distributions over {0,1}*^n^*, the global minimizer *P*^∗^ in Eq. 1 will place all of its mass on the global minimizer of *F*. However, if *T ≫ 0*, the entropy term in Eq. (1) dominates the objective function. With convexity, we can solve a sequence of problems for values of *T*_0_ >*T*_1_ > … >*T*_∞_ = 0 where each of them is initialized at the solution obtained by the previous one. This sequence of temperatures is called as the annealing schedule. When *T* is close to zero the influence of the entropy term becomes shrunken. Therefore, the distribution becomes more concentrated on the minimum of *E_p_*[*F*] which allows us to identify the discrete variables *y* by *p*. Note that there is no guarantee for global optimality because there is not always a path connecting the local minimizers for the chosen sequence of *T* to the global optimum of *F*.

#### Applying DA to SVMs

Given a binary classification problem, we consider a set of *L* training pairs *L* = {(*x*_1_, *y*_1_),…(*x_L_*, *y_L_*)}, *x* ∈ ℝ*^n^*, *y* ∈ {1, –1} and an unlabeled set of *U* test vectors *U* = {*x_L_*_+1_,…,*x_L_*_+_*_U_*} SVMs have a decision function *f_θ_*(·) of the form *f_θ_*(*x*) = *w* · Φ(*x*) + *b*, where *θ* = (*w, b*) are the parameters of the model, and Φ(·) is the chosen feature map, often implemented implicitly using the kernel trick. Given a training set *L* and a test set *U*, for the TSVM optimization problem, find among the possible binary vectors {*γ* = (*y_L_*_+1_,…, *y_L_*_+_*_U_*)} the one such that an SVM trained on *L* ∪(*U* × γ) yields the largest margin. This combinatorial problem can be approximated as finding an SVM separating the training set under constraints which force the unlabeled examples to be as far as possible from the margin. This can be written as minimizing  subject to and . This minimization problem is equivalent to minimizing(6)

where the function *H1*(*·*) = *max*(*0*, *1−·*) is the classical Hinge Loss function. In other words, TSVM seeks a hyperplane *w* and a labeling of the unlabeled examples, so that the SVM objective function is minimized. The discussion in Deterministic Annealing motivates a continuous objective function,(7)

that defined by taking the expectation of *τ*(*f*, *y*′) (Eq. 1) with respect to a distribution *p* on *y*′ and including entropy of *p* as a homotopy term.

For a fixed *T*, the solution to the optimization problem above is tracked as the temperature parameter T is lowered to 0. The DA algorithm returns the solution corresponding to the minimum value achieved when some stopping criterion is satisfied. The criterion used in the DA algorithm is the Pair-wise Mutual Information (PMI) between values of *p* in consecutive iterations. The parameter *T* is decreased in an outer loop until the total entropy falls below a threshold.

### Feature selection

Rich feature sets improve accuracy of the PPI extraction task [27]. The features used in Yang’s paper include word features, keyword features, protein name distance features, and link path features, etc. In this paper, we explore various different features such as syntactic and lexical features as well as semantic features such as negated sentence features, interactor and its POS tag features into the feature sets. The total 9 features were selected for our semi-supervised learning technique (See Table 1).

**Table 1:** Features extracted from example sentence A

Feature	Feature Value
Is negated sentence	True
No. of protein occurrences	3
Interactor name	response
Interactor POS	NN
Interactor position	88
No. of words in between proteins	24
No. of left words	-1
No. of right words	12
Link path status	Yes

***Negation:*** We include whether a sentence is negated or not in the feature set. We use NegEx developed by Chapman and colleagues [28] for negation. NegEx is a regular expression-based approach that defines a fairly extensive list of negation phrases that appear before or after a finding of negation. NegEx treats a phrase as a negated one if a negation phrase appears within *n* words of a finding. The output of NegEx is the negation status assigned to each of the UMLS terms identified in the sentence: negated, possible or actual. NegEx uses the following regular expressions triggered by three types of negation phrases:

*<pre-UMLS negation phrase> {0-5 tokens} <UMLS term> and <UMLS term> {0-5 tokens} <post-UMLS negation phrase>*

There are three types of negation phrases in these expressions: 1) pre-UMLS, 2) post-UMLS and 3) pseudo negation phrases. Pre-UMLS phrases appear before the term they negate, while the post-UMLS phrases appear after the term they negate. Pseudo negation phrases are similar with negation phrases but are not reliable indicators of negation; they are used to limit the negation scope. All UMLS terms inside of the 0-5 tokens window are assigned the negation status depending on the nature of the negation phrase: negated or possible. The example of the negated sentence processed with NegEx is as follows:

*[PREN].No[PREN] relevant changes in heart rate , body weight , and plasma levels of [NEGATED]renin[NEGATED] activity and aldosterone concentration were observed ➔ negated*

***Number of proteins named entities* (*NE*) *occurrences:*** We extracted protein names from each sentence by using a Conditional Random Field (CRF)-based Named Entity Recognition (NER) technique.

To train the CRF NER, we used the training data provided for the BioCreative II Gene Mention Tagging task. The training data consist of 20,000 sentences. Approximately 44,500 GENE and ALTGENE annotations were converted to the MedTag database format [29]. Once we built the train model, we applied the CRF NER to extract proteins or genes from a sentence and counted the number of occurrences of genes in the sentence.

***Interactor:*** Interactor is the term that shows the interaction among proteins in a sentence. The total of 220 interactor terms was identified. We applied a modified UEA stemmer to take care of term variations of interactor [30]. We did not apply an aggressive stemmer like Porter stemmer since we wanted to preserve the POS tag of the interactor.

***Interactor POS:*** As for protein named entities, we applied the CRF-based POS tagging technique to tag tokenized words in a sentence. The CRF-based POS tagger was built on top of the MALLET package [31].

***Interactor position:*** We included the position of the interactor term in a sentence in the feature set.

***Number of words in between proteins:*** We included the number of words in a left most Protein NE and a right most Protein NE in the feature set.

***Number of left words:*** We included the number of words in the left side of the first appearance of a Protein NE in the feature set.

***Number of right words:*** We included the number of words in the right side of the last appearance of Protein NE in the feature set.

***Link path status:*** This feature set is obtained by Link Grammar that was introduced by Lafferty et al. [32]. Link Grammar is used to connect pairs of words in a sentence with various links. Each word is linked with connectors. A link consists of a left-pointing connector connected with a right-pointing connector of the same type on another word. A sentence is validated if all the words are connected. We assume that if a link path between two protein names exists, these two proteins have interaction relation. In our feature selection, if a Link path between two protein names exists, it is set to “Yes”, otherwise, “No”. The Link Grammar parser was used in several papers to extract protein-protein interaction [27,2].

## Results

### Data sets

One of the issues in protein-protein interaction extraction is that different studies use different data sets and evaluation metrics. It makes it difficult to compare the results reported from the studies.

In this paper, we used three different datasets that have been widely used in protein-protein interaction tasks. These are 1) the AIMED corpus, 2) the BioCreAtIvE2 corpus that is provided as a resource by BioCreAtIvE II (Critical Assessment for Information Extraction in Biology) challenge evaluation, and 3) BioInfer corpus. Table 2 summarizes the characteristics of these three datasets.

**Table 2:** Data sets used for experiments

Data Set	Total Sentences	Positive Sentences	Negative Sentences
**AIMED**	4026	951	3075
**BioCreative2**	4056	2202	1854
**BioInfer**	1100	573	527

***AIMED:*** Bunescu et al. [1] manually developed the AIMED corpus3 for protein-protein interaction and protein name recognition. They tagged 199 Medline abstracts, obtained from the Database of Interacting Proteins (DIP) and known to contain protein interactions. This corpus is becoming a standard, as it has been used in the recent studies in several studies [1,15,16].

***BioCreAtIvE2:*** is a corpus for protein-protein interactions, originated from the BioCreAtIvE task 1A data set for named entity recognition of gene/protein names. We randomly selected 1000 sentences from this set and added additional annotation for interactions between genes/proteins. 173 sentences contain at least one interaction, 589 sentences contain at least one gene/protein. There are 255 interactions, some of which include more than two partners (e.g., one partner occurs with full name and abbreviated) [33].

***BioInfer:*** stands for Bio Information Extraction Resource. It was developed by Pyysalo et al. [34]. The corpus contains 1100 sentences from PubMed abstracts annotated for relationships, named entities, as well as syntactic dependencies.

Since previous studies that used these datasets performed 10-fold cross-validation, we also performed 10-fold cross-validation in these datasets and reported the average results over the runs.

For evaluation methodology, we use precision, recall, F-score, and AUC as our metrics to evaluate the performances of the methods.

### Comparison techniques

In this section, we briefly describe other techniques incorporated into semi-supervised SVMs and used to evaluate the performance of active semi-supervised learning models adopted in PPISpotter.

### Baseline: random sampling (RS-SVM)

Random sampling of the unlabeled instances is a naïve approach to semi-supervised learning. We use this approach to compare with the other semi-supervised learning approaches as several studies used this approach to compare it with other semi-supervised learning approaches [19,35].

### Clustering (C-SVM)

One technique is a clustering algorithm applied for the unlabeled data. Fung and Mangasarian [19] used the k-median clustering and showed that the performance was competitive comparing to a supervised learning. The downside of a clustering approach is the correct number of the clusters needs to be pre-defined. We initially tried the two clustering techniques: K-means and Kernel K-means and found that there was only marginal difference in terms of performance. Therefore, we use K-means for the performance comparison.

### Supervised SVMs (SVM)

The kernel we used as the baseline supervised SVM model is a linear kernel. One of the advantages of supervised SVMs with a linear kernel is that it can handle high dimensional data effectively. The reason is it compares the “active” features rather than the complete dimensions. This way, we can impose richer feature sets upon each training example to enhance system performance. The richer feature sets showed to be more effective than the simple feature sets [2]. Another advantage of linear kernel SVM is its low training and testing time costs. In addition, using linear kernel SVM only penalty parameter *C* needs to be adjusted in the algorithm, which is usually set as a constant in applications. In our experiments, the SVM-light package was used. The penalty parameter *C* in setting the SVM is an important parameter since it controls the tradeoff between the training error and the margin. The SVM-light package does an excellent job on setting the default value for this parameter. In our experiments the parameter was left as default value since we observed that other manually determined values of this parameter in fact led to worse performance of supervised SVMs when compared with the default one.

## Discussion

We evaluate and compare the performance of the active semi-supervised machine learning approach (BTDA-SVM) in several different ways. First, we compare it with three different techniques: random sampling, K-means clustering, and supervised SVMs. In addition, we test the performance of BTDA-SVM with supervised counterparts (SVMs) as well as an active learning technique (BT-SVM) for the task of protein-protein interaction extraction. Second, we exam whether the size of combined training datasets between unlabeled and labeled data have impact on the performance. As discussed in Section 3, we Break Tie and Deterministic Annealing, as a kernel function in BTDA-SVM.

Table 3 shows the results obtained with the AIMED data set. Our approach (BTDA-SVM) performs considerably better than other techniques in terms of precision, recall, and F-measure. BTDA-SVM’s performance is superior to the regular SVMs approach by 34.79% in terms of precision. It is 25.55% better than the Random Sampling approach (RS-SVM) in terms of recall. In terms of F-measure, BTDA-SVM is 28.6% better than the regular SVMs. The Break Tie approach (BT-SVM) is the second best in terms of three measures.

**Table 3:** Experimental results – AIMED data set

**Algorithms**	**Measures**		
	
	**Precision**	**Recall**	**F-score**
SVM	55.15%	42.47%	48.14%
RS-SVM	56.98%	41.71%	48.92%
C-SVM	64.53%	40.42%	50.67%
BT-SVM	65.23%	42.51%	53.64%
BTDA-SVM	74.34%	50.75%	61.91%
Yakushiji et al. [15]	33.70%	33.10%	33.40%
Mitsumori et al. [16]	54.20%	42.60%	47.70%

We conducted individual t-tests essentially as specific comparisons. Our prediction that BTDA-SVM would be better than the other comparison techniques (BT-SVM, SVM, RS-SVM, and C-SVM) was confirmed t(11)=3.6966E-11, p<0.05 (one-tailed) at n-1 degrees of freedom (12 runs) while comparing with C-SVM which performed best over the other two comparison techniques. Similarly, the t-test confirmed that the performance difference of BT-SVM is statistically significant from C-SVM t(11)=0.0169, p<0.05 (one-tailed).

In Table 3, we also show the results obtained previously in the literature by using the same data set. Yakushiji et al. [15] used an HPSG parser to produce predicate argument structures. They utilized these structures to automatically construct protein interaction extraction rules. Mitsumori et al. [16] used SVMs with the unparsed text around the protein names as features to extract protein interaction sentences.

Semi-supervised approaches are usually claimed to be more effective when there is less labeled data than unlabeled data, which is usually the case in real applications. To see the effect of semi-supervised approaches we perform experiments by varying the amount of labeled training sentences in the range [10, 3000]. For each labeled training set size, sentences are selected randomly among all the sentences, and the remaining sentences are used as the unlabeled test set. The results that we report are the averages over 10 such random runs for each labeled training set size. We report the results for the algorithms when edit distance based similarity is used, as it mostly performs better than cosine similarity.

**Figure 4:**
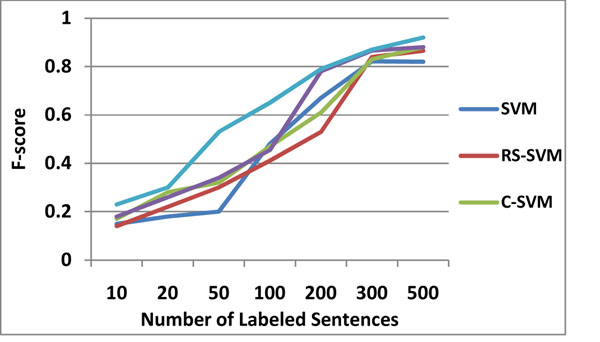
The F-score on the AIMED dataset with varying sizes of training data

**Figure 5:**
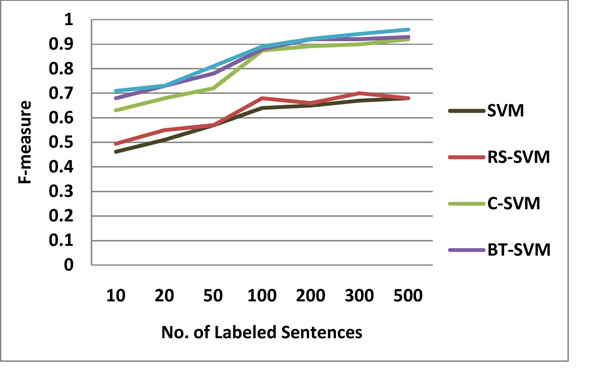
The F-score on the BioCreative II PPI dataset with varying sizes of training data

Figure 4 and 5 show the performance differences of five SVM-based learning techniques as the size of training data increases. BTDA-SVM performs considerably better than their supervised counterpart SVM, RS-SVM, C-SVM when we have small number of labeled training data. It is interesting to note that, although SVM is one of the best performing algorithms with more training data, it is the worst performing algorithm with small amount of labeled training sentences. Its performance starts to increase when number of training data is larger than 200. Eventually, its performance gets close to that of the other algorithms. Harmonic function is the best performing algorithm when we have less than 200 labeled training data.

BTDA-SVM consistently outperforms other techniques in this experiment. We observed that most of the techniques made significant improvement when the training data reaches 200 training instances. Compared to other techniques, BTDA-SVM did not make a radical change to the size of training data.

Table 4 shows the experimental results with the BioCreative2 PPI data set. The performance with BTDA-SVM is always better than other techniques by three measures. BTDA-SVM outperforms the regular SVMs (SVM) by 22.34%, 86.13%, and 48.89% respectively in terms of precision, recall, and F-measure. The second best performance is achieved by BT-SVM in terms of three measures.

**Table 4:** Experimental results – BioCreative2 PPI data set

**Algorithms**	**Measures**		
	
	**Precision**	**Recall**	**F-score**
SVM	70.23%	51.21%	58.33%
RS-SVM	71.7%	56.54%	62.5%
C-SVM	78.23%	88.68%	83.65%
BT-SVM	81.75%	93.5%	85.96%
BTDA-SVM	85.92%	95.32%	86.85%
TSVM-edit [36]	85.62%	84.89%	85.22%

In t-test, we predict that BTDA-SVM would be better than the other three comparison techniques (SVM, RS-SVM, and C-SVM), and the prediction was confirmed t(11)=0.0312, p<0.05 (one-tailed) at n-1 degrees of freedom (12 runs) while comparing with C-SVM. However, our prediction that BT-SVM would be better than C-SVM was not confirmed t(11)=0.092, p<0.05 (one-tailed). As shown in Figure 5, performance curves are different from ones with the AIMED data set. The performance of SVM and RS-SVM is consistently inferior to C-SVM, BT-SVM, and BTDA-SVM.

Although BTDA-SVM consistently outperforms other techniques in this experiment, it does not show statistical significance (In t-test, t(6)=0.2124, p<0.05 (one-tailed) at n-1 degrees of freedom). In addition, all techniques did not make a radical change to the size of training data.

We reported the performance of five comparison techniques with the BioInfer data set. Table 5 shows the experimental results in terms of precision, recall, and AUC. BTDA-SVM’s performance is the best over the other four techniques. It is better than the regular SVMs approach by 25.23%, 19.41%, and 10.32% in terms of precision, recall, and AUC respectively. With respect to AUC, the results of the t-test indicates that BTDA-SVM’s performance is statistically significantly better than the other three comparison techniques (SVM, RS-SVM, and C-SVM), t(11)=8.3483E-6, p<0.05 (one-tailed) at n-1 degrees of freedom (12 runs) while comparing with C-SVM which performed best over the other two comparison techniques. In the same vein, our prediction that BT-SVM would be better than C-SVM was confirmed t(11)=0.00025, p<0.05 (one-tailed).

**Table 5:** Comparison results – BioInfer data set

**Algorithms**	**Measures**		
	
	**Precision**	**Recall**	**AUC**
SVM	65.89%	54.6%	0.843
RS-SVM	64.5%	55.2%	0.847
C-SVM	70.24%	60.2%	0.86
BT-SVM	79.29%	63.1%	0.918
BTDA-SVM	82.52%	65.2%	0.93
Graph Kernel [1]	47.7%	59.9%	0.849

## Conclusions

The goal of our study is two-fold: The first is to explore integrating an active learning technique with semi-supervised SVMs to improve the performance of classifiers. The second is to propose rich, comprehensive feature sets for the protein-protein interaction. To this end, we presented an active semi-supervised SVM-based PPI extraction system, PPISpotter, which encompasses the entire procedure of PPI extraction from the biomedical literature: protein name recognition, rich feature selection, and PPI extraction. In PPI extraction stage, besides several common features such as word features and keyword features, some new useful features including protein names distance feature, phrase negation, and link path feature were introduced for the supervised learning problem. We combined an active learning technique, Break Tie (BT-SVM) with the Deterministic Annealing-based semi-supervised learning technique (DA-SVM), which serves the core algorithm for the PPISpotter system (BTDA-SVM). This BTDA-SVM technique, compared with four different techniques including an active learning technique (BT-SVM), was tested on three widely used PPI corpora. The experimental results indicated that our technique, BTDA-SVM, achieves statistically significant improvement over the other three techniques in terms of precision, recall, F-measure, and AUC.

In future work, we plan to further explore the characteristics of active learning approaches to semi-supervised SVMs and refine our approach to achieve a better PPI extraction performance.

## Competing interests

The author(s) declare that they have no competing interests.

## Authors' contributions

MS developed the fundamental idea of the work, performed experiments, validated the results, and wrote the manuscript. HY and WSH participated in the design, the revision of the study and the experiments.
